# Radcliffe Infirmary, Oxford

**Published:** 1892-05-28

**Authors:** 


### HOSPITAL CONSTRUCTION.

### RADCLIFFE INFIRMARY, OXFORD.

The marked difference of opinion which exists in Oxford at the present time, as to the condition of the Radcliffe Infirmary and the attitude of its critics is sufficiently apparent from the following extracts : The leading paper, the *Oxford Chronicle*, "Trusts that the spirit which appears to animate us (The Hospital) in our criticisms, will be reciprocated by the infirmary authorities." It goes on to show that our case against the Infirmary is practically endorsed by the Committee, who have made "copious quotations from our articles in The Hospital, in the official appeal for subscriptions, and the *Chronicle* expresses the opinion "that they have exercised very considerable effect upon the powers that be." "Confident in the position that 'The Hospital' has taken up, a position which the Infirmary Governors have largely admitted to be just,"—the *Chronicle* urges the Committee to call the city and county together for a public meeting to appoint a special Buildings Committee and a Secretary, and to set vigorously to work to provide the beautiful charity of medicine with a beautiful home at Oxford. The *Oxford Times*, on the other hand, describes our articles as "acrimonious fault finding," and the *Oxford Journal*, "whilst admitting that the authorities of the Oxford Infirmary" have been placed "in a very awkward position," proceeds to speak of "unwarrantable attacks and ridiculous charges." Having done this the *Journal*, with an absence of logic, which is instructive, further proceeds to advocate greater publicity for the appeal and the summoning of a county and city meeting at an early date. There can be no question that that portion of the infirmary building which was condemned long ago by Howard should be taken down and replaced by one which complies with modern rules of hospital construction. As advocating hospital opinion and hospital efficiency we desire to see the Radcliffe Infirmary worthy of our oldest university. Is it too much to hope that the Infirmary Committee and its Governors, will join with us in promptly securing this most desirable result, and that they will afford us an opportunity by energetically and successfully raising this £15,000, to take a hand in the work by subscribing to the extension fund ?

